# Diagnostic capacity, and predictive values of rapid diagnostic tests for accurate diagnosis of *Plasmodium falciparum* in febrile children in Asante-Akim, Ghana

**DOI:** 10.1186/s12936-018-2613-x

**Published:** 2018-12-14

**Authors:** Isabella A. Quakyi, George O. Adjei, David J. Sullivan, Amos Laar, Judith K. Stephens, Richmond Owusu, Peter Winch, Kwame S. Sakyi, Nathaniel Coleman, Francis D. Krampa, Edward Essuman, Vivian N. A. Aubyn, Isaac A. Boateng, Bernard B. Borteih, Linda Vanotoo, Juliet Tuakli, Ebenezer Addison, Constance Bart-Plange, Felix Sorvor, Andrew A. Adjei

**Affiliations:** 1 Department of Biological, Environmental and Occupational Health Sciences, School of Public Health, College of Health Sciences, University of Ghana, Legon, Accra, Ghana; 2 Centre for Tropical Clinical Pharmacology and Therapeutics, School of Medicine and Dentistry, University of Ghana, Accra, Ghana; 3 Department of Molecular Microbiology and Immunology, Johns Hopkins Bloomberg School of Public Health, 615 N. Wolfe St, Baltimore, MD 21205 USA; 4 Department of Population, Family, and Reproductive Health, School of Public Health, University of Ghana, Legon, Accra, Ghana; 5 Department of Health Policy, Planning and Management, School of Public Health, University of Ghana, Legon, Accra, Ghana; 6 Department of International Health, Social and Behavioural Interventions Program, Johns Hopkins Bloomberg School of Public Health, 615 N. Wolfe St, Baltimore, MD 21205 USA; 7 Department of Public and Environmental Wellness, Oakland University, 3101 Human Health Building, 433 Meadow Brook Rd, Rochester, MI 48309-4452 USA; 8 Department of Biochemistry, Cell and Molecular Biology, University of Ghana, Legon, Accra, Ghana; 9 National Malaria Control Programme, Ministry of Health, Accra, Ghana; 10 Asante-Akim Central Municipal Health Directorate, Ghana Health Services, Konongo, Ghana; 11 Regional Health Directorate, Ghana Health Services, Accra, Ghana; 12 Child and Associates, Accra, Ghana; 13 Kpone Katamanso District Health Directorate, Tema, Ghana; 14 Worldwide Universities Network, University of Ghana, P.O. Box LG 13, Legon, Accra, Ghana

**Keywords:** Febrile, Children < 5 years, Rapid diagnostic test (RDT), Malaria, HRP2, Combo

## Abstract

**Background:**

This study seeks to compare the performance of HRP2 (First Response) and pLDH/HRP2 (Combo) RDTs for falciparum malaria against microscopy and PCR in acutely ill febrile children at presentation and follow-up.

**Methods:**

This is an interventional study that recruited children < 5 years who reported to health facilities with a history of fever within the past 72 h or a documented axillary temperature of 37.5 °C. Using a longitudinal approach, recruitment and follow-up of participants was done between January and May 2012. Based on results of HRP2-RDT screening, the children were grouped into one of the following three categories: (1) tested positive for malaria using RDT and received anti-malarial treatment (group 1, n = 85); (2) tested negative for malaria using RDT and were given anti-malarial treatment by the admitting physician (group 2, n = 74); or, (3) tested negative for malaria using RDT and did not receive any anti-malarial treatment (group 3, n = 101). Independent microscopy, PCR and Combo-RDT tests were done for each sample on day 0 and all follow-up days.

**Results:**

Mean age of the study participants was 22 months and females accounted for nearly 50%. At the time of diagnosis, the mean body temperature was 37.9 °C (range 35–40.1 °C). Microscopic parasite density ranged between 300 and 99,500 parasites/µL. With microscopy as gold standard, the sensitivity of HRP2 and Combo-RDTs were 95.1 and 96.3%, respectively. The sensitivities, specificities and predictive values for RDTs were relatively higher in microscopy-defined malaria cases than in PCR positive-defined cases. On day 0, participants who initially tested negative for HRP2 were positive by microscopy (n = 2), Combo (n = 1) and PCR (n = 17). On days 1 and 2, five of the children in this group (initially HRP2-negative) tested positive by PCR alone. On day 28, four patients who were originally HRP2-negative tested positive for microscopy (n = 2), Combo (n = 2) and PCR (n = 4).

**Conclusion:**

The HRP2/pLDH RDTs showed comparable diagnostic accuracy in children presenting with an acute febrile illness to health facilities in a hard-to-reach rural area in Ghana. Nevertheless, discordant results recorded on day 0 and follow-up visits using the recommended RDTs means improved malaria diagnostic capability in malaria-endemic regions is necessary.

## Background

Malaria remains one of the most common causes of febrile illness among people living in tropical and sub-tropical regions. Globally, an estimated 214 million cases were reported which resulted in about 438,000 malaria mortalities [1]. For sub-Saharan Africa (SSA) the malaria burden has continuously been overwhelming, with the incidence of new malaria cases in SSA accounting for 89% of new malaria cases and 91% of malaria deaths in 2015 [1]. While deaths due to malaria have dropped from near 2 million to fewer than 500,000 each year with access to timely diagnosis and effective artemisinin combination therapy, malaria cases have had a less dramatic drop [2]. The goal of a timely malaria diagnosis in Africa is to quickly distinguish life-threatening falciparum malaria from other causes of illness.

Microscopy remains the gold standard for malaria diagnosis because it is inexpensive, has high sensitivity and allows *Plasmodium* species identification and quantification of parasite density [3, 4], however, in rural settings it is often unavailable due to the lack of facilities, expertise and constant power supply. The advent of rapid diagnostic tests (RDTs) for malaria diagnosis is therefore an important development to enhance early diagnosis. The implementation of RDTs has contributed to the timely diagnosis and management of malaria in some endemic countries. There was more than a two-third reduction in anti-malarial drug dispensing for children under-five years upon use of RDTs in some African countries [5, 6].

Although RDTs have simplified the diagnosis of malaria, WHO and other agencies advocate that countries test the sensitivity and specificity of malaria RDTs before giving approval [7–9]. In addition, quality control systems that assure the quality of each batch of RDTs should be implemented through systematic surveillance and monitoring. Where RDTs are available, there is considerable evidence that clinicians treat febrile presentations with anti-malarial drugs, even when the result of the RDT is negative for the presence of the parasite antigen. It is reported that about half of all negative RDT patients were prescribed anti-malarial drugs [10–14]. RDTs for malaria are based on the detection of one of three antigens, histidine-rich protein-2 (HRP2), lactate dehydrogenase (LDH) and aldolase, which distinguish the differences in the sensitivity and specificity seen in RDT test kits [8]. The majority of commercially available malaria RDTs target PfHRP2 [8, 15]. Performance testing of RDTs revealed PfHRP2 to be a more sensitive antigen for detecting *Plasmodium falciparum* infections than other antigens, such as *Plasmodium* lactate dehydrogenase (pLDH) [16]. *Plasmodium falciparum* also produces histidine-rich protein 3 (PfHRP3), an antigen highly similar to PfHRP2 and detected by HRP2-based RDTs [15]; however, PfHRP3 is not detected by all RDTs.

Since their adoption in Ghana, RDTs have contributed to reduction in presumptive treatment [17] but they are not readily available in sufficient quantity even for secondary and referral facilities.

Recent studies in Peru reported field isolates that lack one or both antigens (PfHRP2 and PfHRP3) and that poses a significant problem for diagnosis. Similar findings have also been reported in African countries, including Ghana [18, 19]. This may necessitate the use of HRP2 in combination with other antigens that are more conserved within the parasite (e.g., pLDH or aldolase) [20], to improve diagnostic accuracy.

The aim of the study was to evaluate the implications of a negative malaria test outcome in relation to clinical diagnosis, and to demonstrate the implications of caregiver adherence or otherwise to a negative RDT test in a rural setting in an endemic area. To this end, the diagnostic utility of the HRP2 (First Response), pLDH/HRP2 (Combo), microscopy, and PCR were compared in the following three groups of acutely ill febrile children at presentation (day 0) and follow-up: (i) RDT-positive children who received anti-malarials; (ii) RDT-negative children who received anti-malarials; and, (iii) RDT-negative children who did not receive anti-malarials.

## Methods

### Study sites

This study was conducted in two health facilities (Konongo-Odumase Government Hospital and Juansa Health Centre) in Asante Akim North District of the Ashanti Region of Ghana. The region is one of ten in the country and is located in the Forest Zone where there are two distinct seasons: a wet season (April to October) when malaria transmission is highest and a dry season (December to March). The district occupies an area of 1462 sq km. The main economic activities of the district are subsistence farming, animal husbandry and petty trading.

#### Konongo-Odumase Government Hospital

The hospital provides both general, specialized and referral services to residents in Konongo-Odumase township, surrounding communities and other residents of Ashanti Region. This hospital serves a population of approximately 100,000 with a 50-bed capacity and staff strength of over 250 healthcare providers.

#### Juansa Health Centre

The Juansa Health Centre (JHC), located between Konongo-Odumase and Agogo has a 12-bed capacity, is headed by a physician assistant and provides services to over 15,000 people.

### Study population

The study included all children under 5 years who reported to the health facilities with a history of fever within the previous 72 h or a documented axillary temperature of 37.5 °C.

### Study design

**Fig. 1:**
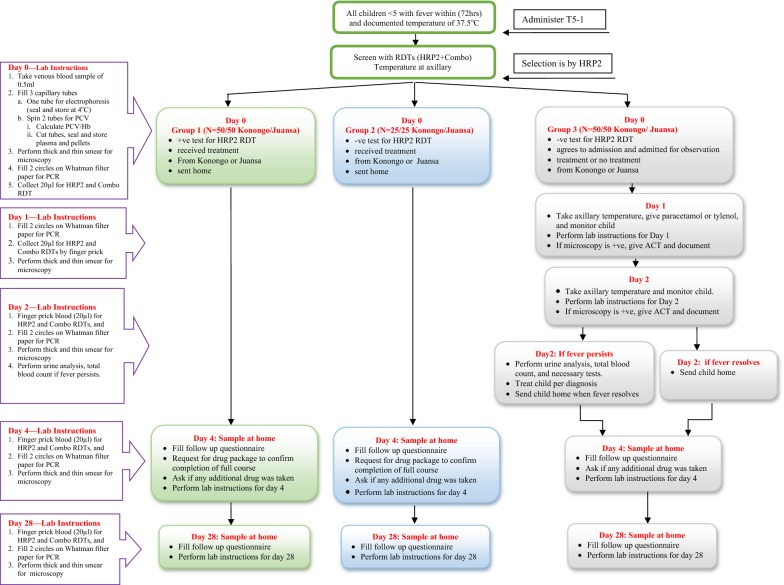
Selection of target patient and associated laboratory instructions: a flow chart

The study, conducted between the months of January and May 2012, employed longitudinal methods that included interventional and quantitative approaches. The sampling strategy and procedures are detailed in Fig. 1. A total of 260 participants were enrolled from the two facilities. Informed consent was obtained from parents/guardians of the children after detailed explanation of the purpose and procedures of the study. Parents/guardians were assisted to complete an interviewer-based, semi-structured questionnaire at the appropriate literacy level.

### Sampling procedure

After obtaining informed consent, the participants were examined by the physician-in-charge, and a study questionnaire was administered to the parent/guardian. This was followed by RDT screening. Based on the results of the RDT and decision of the admitting physician, the children were grouped into one of the following three categories.

- Children < 5 years who presented with fever and tested positive for malaria using RDT and received anti-malarial treatment (Group 1).
- Children < 5 years who presented with fever and tested negative for malaria using RDT and were given anti-malarial treatment by the admitting physician (Group 2).
- Children < 5 years who presented with fever and tested negative for malaria using RDT and did not receive anti-malarial treatment (Group 3).

### Data, sampling and laboratory analysis

Temperature, weight and other demographic characteristics of the children were obtained. Finger and venous blood specimens were collected. All sample collection procedures were done under aseptic conditions. In all, 0.5 µL of venous blood and two dried blood spots (DBS) were deposited onto Whatman 903 protein saver cards with about 50 µL of blood for each circle. The DBS were stored at 20 °C.

### Working principle of First Response^®^ and Combo RDT

The performance of the First Response^®^ Malaria Ag HRP2-HRP2 alone (Premier Medical Corp. Ltd., India) and SD Bioline Malaria Ag Pf/Pan- HRPII and panLDH (Standard Diagnostic Inc. Suwon City, South Korea, Catalogue No: 05FK60) were evaluated following manufacturers’ instructions. Briefly, 20 µL of blood from finger stick was used for the RDTs and colour changes observed after 15 min. For each RDT, cassettes were first labelled with the sample number, then 10 µL of whole blood was added to the sample well and the assay buffer completely emptied into the buffer well. The RDT reaction was considered as positive when two colour bands were seen at the control (C) and test (T) labels. The reaction was considered negative when only one band was seen at the control (C) label. The reaction was considered invalid when no bands were seen at both control and test labels and or when a band was seen at the test label but not at the control label. All invalid reactions were repeated to determine results as either positive or negative.

### Microscopy

Thick and thin blood films were prepared on slides and stained with 10% Giemsa and examined using oil immersion magnification with a light microscope. Two independent microscopists examined slides for asexual parasite stages. Parasite density was quantified in thick films by counting asexual *P. falciparum* parasites against 200 leukocytes and multiplied by 40, assuming a standard leukocyte count of 8000 leukocytes/µL.

### PCR

About 100 µL of blood previously blotted on two circles of Whatman 903 Protein Saver cards filter paper were dried and stored at room temperature (20 °C). Five 3-mm diameter punches were processed with a commercial 96-well kit (Promega, Fitchburg, WI, USA) to extract the DNA from approximately 25 µL of the dried blood into an eluted volume of 200 µL of water. Multiplex PCR from 10 µL (1/20 of 25–1.25 µL of blood equivalents) of the extracted DNA volume was performed in real time (qPCR) on a CFX 384 Detection System Thermocycler (BioRad, Hercules, CA, USA). The thermocycler machine detects 4 probes, therefore *P. falciparum, Plasmodium vivax and Plasmodium malariae* were chosen along with the human actin gene control. The primers and probes were as follows.

*Plasmodium falciparum* 18S rRNA- Forward: 5′-CCACATCTAAGGAAGGCAGCAG Reverse: 5′-CCTCCAATTGTTACTCTGGGAAGG Probe-5′CCCACCATTCCAATTACAA-Cy5.

*Plasmodium vivax* AMA1 Forward 5′-ACGCCAAGTTCGGATTATGG Reverse: 5′-CCGTCATTTCTTCTTCATACTGAG Probe-5′TTGATCTGAGGCACTCGCTCCG-TET.

*Plasmodium malariae* plasmepsin Forward: 5′-CCAACAATACATACACATTAGAACC Reverse: 5′-GTAGGATATAAAGCATACACAAAGTG Probe-5′ATCTAGTAATGGCTCC-TX Red Human beta actin For 5′-GTGCTCAGGGCTTCTTGTCC Rev 5′-CCATGTCGTCCCAGTTGGT Probe-5′ACCCATGCCCACCATCACGCCC-FAM.

The human actin gene was used as an extraction control and PCR was performed in duplicate from the single extraction of each sample.

A cycle count of 34 was used for the cut off to separate positive and negative PCR samples. The efficiencies for the amplifications were 150% for *P. falciparum*, 101% for *P. malariae* and 70% for *P. vivax*.

### Case definition

True positives (TP) for RDT were defined as PCR positive and/or microscopy positive. False positives (FP) were cases in which PCR and microscopy negatives were positive for RDT. True negatives (TN) were negative by all three methods. False negatives (FN) were those cases that were negative by RDT but positive for PCR and/or microscopy.

### Counselling and follow-up of patients with initial negative and positive results

Follow-ups were done by registered nurses and was coordinated by research assistants, the biomedical scientist at Konongo, and the Municipal Director of Health Services. Children who tested positive by RDT and received anti-malarials (Group 1) were followed up as outpatients on day 4 and day 28 in their homes. Children who tested negative and received anti-malarials (Group 2) were also followed up as outpatients on day 4 and day 28 at their respective homes. Children who tested negative and did not receive anti-malarials (Group 3) were placed under observation overnight. The plan for observing and following up of participants is detailed in Fig. 1.

### Statistical analysis

Data were entered into spreadsheets using Microsoft Excel and analysed with the Statistical Package for Social Sciences (SPSS) version 17 (SPSS Inc., Chicago, IL, USA). Simple descriptive statistics were used to analyse the demographic data. The malaria parasite density was log transformed before analysis. Significant levels were measured at 95% confidence intervals and values were considered significant at P < 0.05. Sensitivity and specificities of the tests were calculated from the TP, TN, FP, and FN test results using the formulae below.

Sensitivity = TP/(TP + FN), Specificity = TN/(TN + FP), Positive predictive value = TP/(TP + FP), Negative predictive value = TN/(TN + FN). The values obtained were expressed as percentages by multiplying by 100.

## Results

### Characteristics of study participants

A total of 260 children < 5 years reporting with fever were recruited from the Konongo-Odumase Government Hospital and Juansa Health Centre, both in the Asante-Akim North District. The mean age was 22 months and females accounted for nearly 50% (49.8%) of the study participants. At the time of diagnosis, the mean body temperature was 37.9 °C (range 35–40.1 °C).

### Comparison of microscopy, qPCR, HRP2, Combo RDTs

Tables 1 and 2 show the results of all four diagnostic methods deployed in the study: HRP2-RDT 32% (83/260), Combo-RDT 31% (81/260), microscopy 31% (81/260), and qPCR 38% (98/259). Microscopic parasite density ranged between 300 and 99,500 parasites/µL (Table 1). Thin blood film showed *P. falciparum* in all blood specimens except three individuals who were positive for *P. malariae* (two of which were mixed with *P. falciparum*). None was positive for *P. vivax* by qPCR; *P. falciparum* schizonts were observed in one sample. No gametocytes were detected at the microscopic level.

**Table 1:** Comparison of rapid diagnostic test (RDTs) HRP2 (First Response^®^) and pLDH/HRP2 (Combo^®^) with microscopy

Tests results	GS: Microscopy^a^ (n = 260)		Prevalence	Sensitivity (95% CI)	Specificity (95% CI)	PPV (95% CI)	NPV (95% CI)
	Positive	Negative					
HRP2							
Positive	77	6	31.3 (25.7–37.3)	95.1 (87.8–98.6)	96.6 (92.8–98.8)	92.8 (85.4–96.6)	97.7 (94.3–99.1)
Negative	4	173					
Combo							
Positive	78	3	31.2 (25.9–37.2)	96.3 (89.6–99.2)	98.3 (95.2–99.7)	96.3 (89.4–98.8)	98.3 (95.1–99.4)
Negative	3	176					

*GS* gold standard, *PPV* positive predictive value, *NPV* negative predictive value, *HRP2* histidine-rich protein 2, *pLDH* lactate dehydrogenase

^a^Mean parasite density 29,721.6/µL (300–99,500/µL), Trophozoites 50, Schizont 1

**Table 2:** Comparison of rapid diagnostic test (RDTs) HRP2 (First Response^®^) and pLDH/HRP2 (Combo^®^) and microscopy with PCR

Tests results	GS: PCR (n = 246)		Prevalence	Sensitivity (95% CI)	Specificity (95% CI)	PPV (95% CI)	NPV (95% CI)
	Positive	Negative					
HRP2							
Positive	72	10	39.8 (33.7–46.3)	73.5 (63.6–81.9)	93.2 (87.9–96.7)	87.8 (79.6–93.0)	84.2 (79.2–88.1)
Negative	26	138					
Combo							
Positive	71	9	39.8 (33.7–46.3)	72.5 (62.5–81.0)	93.9 (88.8–97.2)	88.6 (80.5–93.8)	83.7 (78.8–87.7)
Negative	27	139					
Microscopy							
Positive	70	9	39.8 (33.7–46.3)	71.4 (61.4–80.1)	93.9 (88.8–97.2)	88.6 (80.3–93.7)	83.2 (78.4–87.2)
Negative	28	139					

*GS* gold standard, *PCR* polymerase chain reaction, *PPV* positive predictive value, *NPV* negative predictive value. *HRP2* histidine-rich protein 2, *pLDH* lactate dehydrogenase, *CI* confidence interval

There were ten negative samples for qPCR, which were positive for RDTs and microscopy. With microscopy as gold standard, the sensitivity of HRP2 and Combo-RDTs was 95.1 and 96.3%, respectively. The sensitivities, specificities and predictive values for RDTs were relatively higher in microscopy-defined malaria cases than in qPCR positive-defined cases.

### Microscopy and Combo results of HRP2-negative febrile children during 28-day follow-up

#### HRP2-negative children not treated with anti-malarials

All febrile children who were initially HRP2-negative (n = 95) and did not receive anti-malarials were followed up. Day-0 results of initially HRP2-negative children found later to be positive were microscopy (n = 2), Combo (n = 1) and PCR (n = 17) (Table 3). On days 1 and 2, five of the children in this group tested positive by PCR alone. On day 4, children who were originally HRP2-negative tested positive for microscopy (n = 1) and Combo (n = 1) (Table 3). On day 28, four patients who were originally HRP2-negative tested positive for microscopy (n = 2), Combo (n = 2) and PCR (n = 4) (Table 3). A child in Group 3 was positive for all three malaria tests on day 4, whereas three were positive only for PCR. On day-28 follow-up, two children were positive for all three tests, whereas four children were positive for PCR (Table 3). It is noteworthy that all children in this group who initially tested negative by HRP2 and later tested positive with microscopy at follow-up, were treated with an appropriate anti-malarial and dropped out of the group. More so, children in this group who tested positive on days 4 and 28 were referred for further management.

**Table 3:** Follow-up results for all tests (microscopy, PCR and Combo) after initial testing with HRP2

Follow-up	Test	Group 1	Group 2	Group 3
Day 0	Microscopy	67	1	2
	HRP2	72	0	0
	Combo	70	0	1
	PCR	72	9	17
Day 1	Microscopy	–	–	0
	HRP2	–	–	0
	Combo	–	–	0
	PCR	–	–	5
Day 2	Microscopy	–	–	0
	HRP2	–	–	0
	Combo	–	–	0
	PCR	–	–	5
Day 4	Microscopy	5	1	1
	HRP2	5	1	1
	Combo	5	1	1
	PCR	10	0	3
Day 28	Microscopy	2	3	2
	HRP2	0	0	2
	Combo	0	0	2
	PCR	8	2	4

#### HRP2-negative children treated with anti-malarials

In this group (n = 68) (children < 5 years who presented with fever and tested negative using RDT and received anti-malarial treatment), one child tested positive by microscopy, and nine children tested positive by PCR on day 0.

#### HRP2-positive children treated with anti-malarials

A total of five children in this group tested positive on day 4 for HRP2, microscopy and Combo tests and 10 by PCR. However, on day 28, two children were positive by microscopy and eight by PCR.

## Discussion

Malaria remains a major public health problem in many countries. In the quest to effectively manage cases, early diagnosis and prompt treatment with efficacious anti-malarials is advocated [21]. The WHO recommends all patients receive parasitological confirmation by microscopy or RDTs before malaria treatment begins [7]. However, although RDTs are a good alternative to microscopy in resource-poor settings, RDTs cannot quantify the parasite load and are ineffective for diagnosing recently treated individuals.

The results indicate that both HRP2 and Combo RDTs recorded high sensitivity when microscopy was used as gold standard. These sensitivity rates are comparable with reports from previous studies [22], but higher than reported by Sani et al. [23] in Nigeria. It is noteworthy that both sensitivity and specificity values for HRP2 and Combo RDTs in this study meet the minimal standard of 95% for *P. falciparum* [9]. Most commercially available RDTs detect PfHRP2 alone or a combination of PfHRP2 and pLDH. The choice of PfHRP2 is influenced among others by its specificity to the predominant cause of malaria, *P. falciparum*. In endemic areas, it is also characteristic for HRP2 antigen to be produced at the asexual and early gametocyte stages of *P. falciparum* life cycle [24], and its persistence possibly explains the false positives recorded [25, 26]. In addition, HRP2 antigens are produced by the schizonts at an early stage of the parasite, even before the parasites are initially released into peripheral circulation [22], while pLDH is more conserved and is cleared after a relatively shorter period. Indeed, it has recently been shown that a large proportion of children (up to 25%) treated for malaria based on positive HRP2-RDT results were children who were not infected with malaria, if microscopy is taken as the gold standard [27]. Aside from HRP2 persistence, other possible reasons for false-positive results include non-specific bindings or inference with other immunological or infectious factors [28–31].

Moreover, the sensitivity of the HRP2 (First Response^®^ Malaria Kit) recorded in this study contrasts with that reported by Ndamukong-Nyanga et al. (95 vs 48.5%) in Cameroon. For the Combo RDT, Xiaodong et al. [32] reported < 90% sensitivity, which is comparable to sensitivity found in the present study. The positive predictive value (PPV) and negative predictive values (NPV) for the HRP2 and Combo were comparable and both > 92% are higher than those reported by others elsewhere for HRP2 (a PPV of 62.3% and NPV of 75% [33] and for Combo RDT, PPV of 38.3% and NPV of 14.3% [34].

When PCR was used as reference, HRP2 and Combo RDTs recorded lower sensitivity, specificity, PPV, and NPV (Table 3). The higher accuracy by the more sensitive PCR may be indicative of false-negative RDT results as is often seen in patients with low parasitaemia [35, 36]. However, false-negative RDT results have also been reported at high densities, due to the prozone phenomenon in HRP2-based RDTs [37], suggesting the continuous need for alternative diagnostic markers for effective screening that are more predictive in field application and suitable for point-of-care application, where resources and expertise to perform advanced laboratory diagnostics are unavailable.

Some recent studies have reported an increase in false-positive results of HRP2-based RDTs due to mutations in the antigen [19, 38, 39]. Some parasite strains from Africa and South America have been reported to lack the HPR2 antigens [19, 38–41].

In determining which markers are best diagnostic preferences for malaria in RDTs, some studies that compare RDT *versus* microscopy tend to use PCR as a confirmatory test. The sensitivity, specificity and predictive values of the Combo RDT were higher than the HRP2 with PCR as the gold standard. In view of the fact that HRP2-based RDTs are more sensitive than LDH-based RDTs at low parasite densities, the findings are in agreement with the general conclusions that a positive LDH RDT suggests a parasite density above HRP2 detection threshold [16], while microscopy-positive LDH-negative samples may reflect low density infections.

The use of RDTs can reduce overprescribing of anti-malarial drugs, and studies have shown that health workers prescribe anti-malarials to patients with negative RDT results [42]. In endemic areas, the presence of malaria parasites in blood may not necessarily reflect a clinical malaria episode [43], while non-compliance to RDT-negative results by prescribing anti-malarial drugs may neglect an underlying infection. Factors associated with compliance to negative RDT results include trust in the result and knowledge of alternative diagnosis [14, 44], both of which could be enhanced by improving diagnostic capacity for other common febrile illnesses and by developing evidence-based guidelines for treatment of symptomatic RDT-negative patients [42].

The sensitivity of HRP2 and pLDH as diagnostic markers in *P. falciparum* has shown a sensitivity of about 95.2 and 98.5%, respectively, from previous works [45, 46] and this is similar to the results reported herein (Tables 1, 2 and 3).

A major limitation to this study is that the authors were unable to perform any genotyping or sequencing on samples collected on various follow-up visits. It is therefore not possible to draw definitive conclusions as to whether seropositivity for malaria parasites, antigens/DNA was due to a persistent infection or to new infections.

## Conclusion

HRP2- and pLDH-based RDTs showed comparable diagnostic accuracy in children presenting with an acute febrile illness to health facilities in a hard-to-reach rural area in Ghana. However, the presence of discordant results between the recommended diagnostic tests on presentation and during follow-up suggest the need for improving diagnostic capability for febrile illness in malaria-endemic areas.
